# Infection Meets Inflammation: N6-Methyladenosine, an Internal Messenger RNA Modification as a Tool for Pharmacological Regulation of Host–Pathogen Interactions

**DOI:** 10.3390/biom13071060

**Published:** 2023-06-29

**Authors:** Milena N. Leseva, Brigitta Buttari, Luciano Saso, Petya A. Dimitrova

**Affiliations:** 1 Laboratory of Experimental Immunotherapy, Department of Immunology, The Stephan Angeloff Institute of Microbiology, Bulgarian Academy of Sciences, 1113 Sofia, Bulgaria; petya_dimitrova@web.de; 2 Department of Cardiovascular and Endocrine-Metabolic Diseases and Aging, Istituto Superiore di Sanità, 00161 Rome, Italy; brigitta.buttari@iss.it; 3 Department of Physiology and Pharmacology “Vittorio Erspamer”, La Sapienza University of Rome, 00185 Rome, Italy; luciano.saso@uniroma1.it

**Keywords:** epitranscriptome, N6-methyladenosine, m6A, host–pathogen interactions, immune cells, infectious disease, viral infection

## Abstract

The significance of internal mRNA modifications for the modulation of transcript stability, for regulation of nuclear export and translation efficiency, and their role in suppressing innate immunity is well documented. Over the years, the molecular complexes involved in the dynamic regulation of the most prevalent modifications have been characterized—we have a growing understanding of how each modification is set and erased, where it is placed, and in response to what cues. Remarkably, internal mRNA modifications, such as methylation, are emerging as an additional layer of regulation of immune cell homeostasis, differentiation, and function. A fascinating recent development is the investigation into the internal modifications of host/pathogen RNA, specifically N6-methyladenosine (m6A), its abundance and distribution during infection, and its role in disease pathogenesis and in shaping host immune responses. Low molecular weight compounds that target RNA-modifying enzymes have shown promising results in vitro and in animal models of different cancers and are expanding the tool-box in immuno-oncology. Excitingly, such modulators of host mRNA methyltransferase or demethylase activity hold profound implications for the development of new broad-spectrum therapeutic agents for infectious diseases as well. This review describes the newly uncovered role of internal mRNA modification in infection and in shaping the function of the immune system in response to invading pathogens. We will also discuss its potential as a therapeutic target and identify pitfalls that need to be overcome if it is to be effectively leveraged against infectious agents.

## 1. Introduction

**Figure 1:**
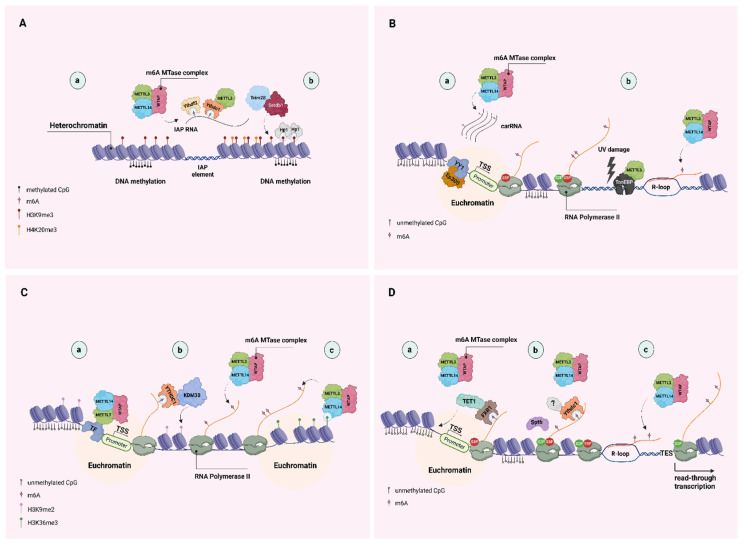
M6A as a global regulator of the chromatin state, genomic stability, and transcription. Figure created with BioRender.com (**A**). Mettl3-mediated m6A maintains the heterochromatin state. a—Mettl3 localizes to the intracisternal A particle (IAP) family of endogenous retroviral elements (ERVs). M6A modification of IAP mRNA reduces its half-life and promotes its degradation following the binding of Ythdf2, which contributes to the suppression of ERVs [12]. b—Mettl3 recruits Setdb1 and Trim28 to IAP regions. This results in H3K9 tri-methylation, which is reinforced by Hp1 binding and H4K20me3—both histone modifications are important for the repression of repetitive elements under normal conditions [13]. (**B**) Mettl3-mediated m6A maintains genomic stability. a—Mettl3 depletion, and the consequent m6A reduction, leads to an accumulation of carRNAs (promoter-associated, enhancer, and repeat RNA), which promotes local chromatin accessibility and downstream transcription of protein-coding genes. carRNA with reduced m6A is associated with the enhanced recruitment of the transcriptional regulators Yy1/Ep300 [14]. b—UV-induced DNA damage is sensed by TonEBP, which directly recruits Mettl3 to R-loops. M6A modification followed by downstream RNaseH1-dependent resolution of the R-loop maintains the genomic integrity [15]. (**C**) Cross-talk between m6A, transcriptional regulators, and epigenetic modifications. a—The core components of the m6A RNA methyltransferase complex (MTase) can be recruited to promoter regions by transcription factors (TF) [16]. b—M6A is placed co-transcriptionally on mRNA and is recognized by YTHDC1, which recruits the H3K9me2 demethylase, KDM3B, and promotes gene expression [17]. c—H3K36me3, a marker for transcriptional elongation, recruits the m6A MTase complex through direct interaction with Mettl14 and facilitates co-transcriptional m6A deposition [18]. (**D**) M6A as a regulator of transcriptional activity. a—M6A recruits the reader protein FXR1, which interacts with the methylcytosine dioxygenase TET1 to demethylate DNA, thus, promoting chromatin accessibility and gene expression [19]. b—In *Drosophila*, m6A regulates promoter-proximal pausing of RNA Polymerase II (RNAP II). Mettl3-mediated m6A is required to release RNAP II into the gene body for productive transcription. Ythdc1 recognizes m6A and interacts, through an unknown intermediate, with the histone chaperone and transcription elongation factor Spt6 [20]. c—In R-loops formed around transcriptional end sites (TES), RNA is m6A-modified. Depletion of Mettl3 leads to a significant reduction in R-loop levels, which results in defective RNAP II termination and read-through transcription downstream of the TES [21]. *Abbreviations:* MTase—methyltransferase complex; IAP—intracisternal A particle; carRNA—chromosome-associated regulatory RNA; Ythdf2—YTH domain family member 2; Ythdc1—YTH domain containing protein 1; Setdb1—SET domain bifurcated histone lysine methyltransferase 1; Trim28—Tripartite motif containing 28; Hp1—Heterochromatin protein 1; Ep300—E1A binding protein p300; Yy1—Yin and Yang protein 1; UV—ultraviolet; TonEBP—Tonicity responsive enhancer binding protein; KDM3B—Lysine demethylase 3B; FXR1—Fragile X mental retardation autosomal homolog 1; TET1—Tet methylcytosine dioxygenase 1; Spt6—Suppressor of Ty 6; TSS—transcription start site; TES—transcription end site; RNAP II—RNA polymerase II; S5P—RNAP II phosphorylated on the C-terminal domain in the serine 5 position; S2P—RNAP II phosphorylated on the C-terminal domain in the serine 2 position; H3K9me3—trimethylation of lysine 9 on histone H3; H3K9me2—dimethylation of lysine 9 on histone H3; H3K36me3—trimethylation of lysine 36 on histone H3; H4K20me3—trimethylation of lysine 20 on histone H4; m6A—N6-methyladenosine.

The chemical modification of RNA nucleosides was first described back in the 1970s [1,2,3,4,5]. The 1990s saw the purification of the first RNA modifying enzymes and cloning of their respective genes [6,7]. The role of modified RNA in suppressing innate immune cells was uncovered in the early 2000s when, for the first time, a physiological function for naturally occurring modified RNA nucleosides was proposed [8]. Recently, there has been an uptick of interest into the characterization of the many modifications (now >150) that occur on cellular transcripts, collectively referred to as the *epitranscriptome*. Advances in sequencing technologies [9]—from immunoprecipitation-based methods with limited resolution using modification-specific antibodies to single-nucleotide resolution approaches—have allowed us to map the more abundant modifications (e.g., N6-methyladenosine, 5-methylcytosine, and N1-methyladenosine) on protein-coding and non-coding RNAs. We are able to identify the preferred sequence motifs in which they occur, to quantify the modified transcripts at the transcriptome level, and to examine the biological functions of RNA modifications [10]. More recently, we also strove to improve our understanding of modification stoichiometry and its dynamics [11]. For example, it was shown long ago that there are ~3 N6-methyladenosines per poly(A) transcript in mammalian cells [1]—or one m6A peak per 2000 nucleotides [10]—however, this is a number averaged across all transcripts of a gene, not a direct measurement on a particular transcript. The improved ability to detect modifications on single transcripts will allow us to fully appreciate their effects on RNA metabolism and will help reveal as yet unknown functions. The ongoing discovery of proteins that reverse modifications underscores their dynamic regulation in the cell, which can depend on internal factors and/or external signals. RNA modifications serve as an additional layer of post-transcriptional gene expression regulation influencing mRNA export from the nucleus, its localization in the cytoplasm, secondary structure, stability/half-life/decay, and translational efficiency. However, it appears that is not all. The roles of RNA modification, particularly for methylation at N6-adenosine, in the context of the chromatin environment, were more recently described, and they are summarized in Figure 1.

## 2. M6A Modification—A Dominating Internal Modification with Far-Reaching Implications

N6-methyladenosine (m6A) is the most abundant epitranscriptomic mark in mammals, representing 0.11–0.5% [22,23] of all adenosines in poly(A) RNA (measured by quantitative mass spectrometry), and as such, dominates the current RNA modification literature. The writer complex that sets this mark is a large 1 MDa holocomplex consisting of the core heterodimer methyltransferase 3/14 (METTL3/METTL14) and the accessary proteins Wilms’ tumor 1-associated protein (WTAP), Vir like m6A methyltransferase associated (VIRMA), RNA-binding motif protein 15 (RBM15A/B), zinc finger CCCH domain-containing 13 (ZC3H13), and Cbl proto-oncogene like 1 (CBLL1/HAKAI) [24]. METTL3 is responsible for the m6A catalytic activity, and its knockout in mice is embryonically lethal in the early post-implantation stage (E6.5) as a result of the failure to downregulate the naïve pluripotency program [25]. The knockout of METTL14 is also lethal early in development [26], highlighting the importance of m6A for proper embryogenesis and cell-fate commitment in mammals. This modification is set co-transcriptionally and occurs in the DRACH consensus sequence motif [D = A/G/U, R = A/G, H = A/C/U]. While DRACH is not specifically enriched at any particular site along the length of the transcripts, m6A is consistently shown to occur predominantly at long internal exons, 3′-exons, near stop codons, and within 3′UTRs. The nature of this specificity remained unexplained for a long time. Two recent studies [27,28] point to the importance of the exon junction complex (EJC) in shaping m6A distribution profiles. Yang et al. demonstrated that the core RNA-binding component of the eukaryotic translation initiation factor 4A3 (EJC-EIF4A3) restricts m6A modification levels at both coding and long non-coding transcripts, an effect specific to short internal exons and exon–exon boundaries [27]. The knockdown of EIF4A3 in HeLa cells significantly increases m6A at short internal exons, and, while no such effect is observed at long exons, it elevates m6A methylation at sites near splice junctions. EIF4A3 depletion was associated with an increase in m6A levels at splice sites and with alternative exon usage, and it, especially, affected long multi-exon RNAs. Mechanistically, the authors demonstrated an increased recruitment of METTL3 to sites that were hypermethylated for m6A upon EIF4A3 knockdown [27]. In their study, Uzonyi et al. challenged the paradigm of selective m6A deposition and suggested that the modification was placed by default on its consensus sequence motifs [28]. The authors demonstrated a high degree of agreement between the experimentally measured m6A and their prediction model based on only two considerations: that every eligible m6A site is methylated and that this occurs unless the site is within an exclusion-zone of ~100 nucleotides from an exon/intron junction; a model of m6A deposition is termed *exclusion-based* [28]. This study found that exon density inversely correlates with both measured and predicted m6A levels and, conversely, that there is a positive correlation between exon density and mRNA half-life. The latter correlation was lost upon METTL3 knockout or inhibition, suggesting that m6A functionally connects RNA exon–intron architecture with RNA decay [28]. Collectively, these studies proposed that the EJC, and in particular EIF4A3, can physically block METTL3-mediated m6A deposition and is responsible for its observed pattern of distribution.

The m6A modification is regulated by the stability and enzymatic activity of the METTL3/METTL14 heterodimer, supported by the accessory proteins. In particular, WTAP is critical for enzyme activity and is an essential component of the methyltransferase complex. However, m6A can occur at WTAP-dependent as well as WTAP-independent sites [29]. While WTAP-dependent sites are key for the regulation of “basal” mRNA degradation and are located at internal positions in the transcript, WTAP-independent sites are characteristic for their cap structure [29]. Other accessory proteins act differently on METTL3/METTL14 and affect m6A deposition via guidance of the complex to select sites. For example, VIRMA recruits the complex to regions in the 3′UTR and near stop codons and regulates the polyadenylation and length of several hundred transcripts [30]. Accessory proteins are important for the function of the METTL3/METTL14 complex, however, other regulators oppose the methylation process. This mechanism includes the removal of m6A by two enzymes from the AlkB (α-ketoglutarate dependent dioxygenase) family-ALKBH5 (AlkB homolog 5) and FTO/ALKBH9 (fat mass and obesity-associated protein). This indicates that the expression of Mettl3 and Mettl14, the assembly of the methyltransferase complex, the presence and abundance of accessory proteins, and the expression and activity of demethylases are all critical for m6A maintenance and act in a cell-type-specific manner during cellular differentiation and the execution of effector functions.

## 3. RNA Modification in Regulation of Immune Cell Homeostasis, Differentiation and Function

### 3.1. Models of Hematopoiesis

**Figure 2:**
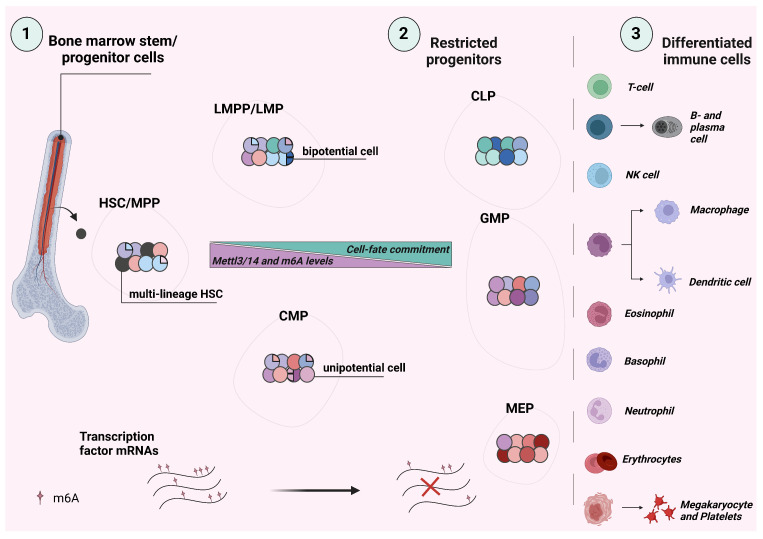
M6A dynamics during adult hematopoiesis in the bone marrow. Figure created with BioRender.com. Schematic representation of adult hematopoiesis with colored circles depicting single stem and progenitor cells that occupy compartments (grey boundaries), which are organized in a hierarchical manner. These cell populations are phenotypically heterogeneous, and alternative routes to their downstream differentiation can exist within each compartment. Black circles depict multipotent cells with stem cell characteristics; single colored circles depict cells with uni-lineage potential, while circles with two colors represent bi-potential cells. As their lineage bias increases from left to right, cells gradually transition from stem cells to primed to restricted progenitor cells, then commit to their respective lineage and end up as differentiated immune cells with specific functions. Color coding is used to visualize these differentiation trajectories. Differentiation coincides with a decrease in expression levels of Mettl3 and Mettl14, as well as a respective decrease in global m6A levels. The m6A modification can mark transcripts for transcription factors with essential functions during hematopoiesis (e.g., lineage-specification and proliferation) and can potentially function as a mechanism to stabilize transcripts or target them for degradation or affect the efficiency with which they are translated, thereby affecting transcription factor expression levels. Abbreviations: HSC—hematopoietic stem cell; MPP—multipotent progenitor; LMPP—lymphoid-primed multipotent progenitor; LMP—lymphoid–myeloid progenitor; CMP—common myeloid progenitor; CLP—common lymphoid progenitor; GMP—granulocyte–monocyte progenitor; MEP—megakaryocyte-erythroid progenitor.

Hematopoiesis is a tightly regulated process in the bone marrow through which mature blood cells are generated. It entails the progressive commitment of hematopoietic stem cells (HSC) to multipotent progenitors through cell division and then to restricted progenitors, which follow their lineage trajectories to terminally differentiated cells with specific functions (reviewed by E. Laurenti and B. Göttgens, 2018) [31]. The dysregulation of hematopoiesis underlies many immune-mediated diseases. According to the classical model, lineage commitment and differentiation take place at stages, in which stable progenitor populations—defined by specific combinations of cell surface markers and involving particular transcription factors—progress from multi- to oligo- to uni-potent cell states in a switch-like manner. In particular, HSCs can commit to the LSK compartment of Lin^−^/Sca-1^+^/c-Kit^+^ cells for distinct multipotent progenitor generations (MPP1 through 5), each with self-renewal potential [32]. Despite the fact that this model is validated by various studies showing the pivotal role of transcription factors in drawing lineage trajectories, transplantation experiments, single-cell data, and transcriptomic and proteomic analyses have demonstrated that hematopoiesis is far more plastic and flexible (reviewed by M. Brand and E. Morrissey, 2020) [33]. For example, megakaryocytes may originate either directly from HSCs or from megakaryocyte/erythroid progenitors (MEPs); basophils arise from granulocyte–macrophage progenitors (GMPs) and also from a branch of the erythroid-megakaryocyte lineage; monocytes can originate from monocyte–dendritic progenitor (MDP) and granulocyte–monocyte progenitor (GMP) lineages [33]. Recently, a new model of hematopoiesis was proposed (Figure 2). According to it, hematopoiesis is progressive but not switch-like and has a constant differentiation landscape where the loss of cell-fate potential and lineage commitment of hematopoietic progenitors is smooth, and bi-potential progenitors are not clearly defined or static [31,33]. The extracellular environment of the hematopoietic stem cell niche provides the variable levels of restriction that alter cell-fate probabilities in all progenitors, driving their differentiation at specific locations [34].

### 3.2. M6A in Hematopoietic Stem Cell/Progenitor Cell Differentiation

HSCs, progenitors, and terminally differentiated cells have various levels of the m6A modification that correspond to their specific self-renewal potential, proliferation state, and transcriptional activity. Methylation of mRNA occurs during the commitment to particular lineage trajectories, when m6A can regulate mRNA abundance for pivotal transcription factors and regulators of the developmental program and/or cell fate. The largest changes in mRNA abundance in each cell type occur when transcripts have accumulated m6A in clusters (reviewed by AM Heck and CJ Wilusz, 2019) [35]. Transcripts with single m6A modified sites may change more gradually, and transcripts that lack m6A are the slowest to change [35]. This mechanism can increase/decrease the activity of cellular pathways above or below a certain threshold needed for cell commitment with or without proliferation or, conversely, to maintain self-renewal or quiescence. While the master regulators of differentiation can initiate the transition to more differentiated states through elevated mRNA abundance, increased m6A methylation of transcripts for other cellular proteins can help coordinate and fully stabilize this transition towards the differentiated phenotype. Thus, the loss of m6A methylation is associated with elevated self-renewal potential and inhibition of cell commitment, especially at early developmental stages [35]. Another important role for m6A in this context might be related to the clearance of existing transcripts through recruitment of the mRNA decay-promoting factor, the reader protein YTHDF2. This mechanism supports the new model of hematopoiesis, where cells in transition do not proliferate yet are still committed to the lineage trajectories. M6A can induce clearance of mRNAs at a specific developmental transition stage and can also facilitate transcriptional pre-patterning, where mRNAs for an alternative cell fate are actively being transcribed but are constantly targeted for m6A-mediated decay before their translation (Figure 2) [36].

The main driver of hematopoiesis is the activation of specific transcriptional programs, however, there is accumulating evidence for the role of RNA modifications, specifically m6A, in hematopoietic stem cell differentiation. Using a single-nucleotide resolution approach for m6A quantification (m6A-SAC-seq), Hu et al. [11] recently showed that, during in vitro differentiation of human umbilical cord-blood derived CD34^+^ HSPC cells (hematopoietic stem/progenitor cells), many regulators of hematopoiesis have transcripts modified by m6A (e.g., *RUNX1*, *FOS*, *ZEB*, *ETV6*, *XIST*, *MALAT1*). Interestingly, transcripts for *METTL3/14*, *WTAP*, the m6A reader proteins *YTHDF1/2/3,* and the demethylase *ALKBH5* are also modified, suggesting the potential for auto-regulatory feedback loops. The authors also reported a surprisingly dynamic m6A-methylome during differentiation, with many m6A sites at CDS, introns, and 3′UTRs either lost or gained, compared to the previous time-point (days; d0-d3-d6-d9). Gene ontology analysis showed that genes involved in cellular functions such as cell cycle regulation and DNA damage and repair were subjected to more dynamic changes in m6A stoichiometry, whereas genes involved in immune response, myeloid activation, RNA splicing, and catabolic processes had more stable m6A stoichiometry [11].

One of the first insights into the role of m6A during hematopoiesis came from Weng et al., who demonstrated that in the mouse bone marrow, *Mettl14* is highly expressed in HSC and LSK cells (Lin^−^/Sca-1^+^/c-Kit^+^); it is downregulated in hematopoietic progenitor cells and common myeloid progenitors (CMPs) while remaining high in megakaryocyte/erythroid progenitor cells (MEPs) [37]. The *Mettl14* expression level was decreased in granulocyte/macrophage progenitors (GMPs) and lowest in Mac1^+^/Gr-1^+^ cells, suggesting that Mettl14 and m6A are downregulated during myeloid differentiation (Figure 2) [37]. A similar pattern of expression for *Mettl3* and *Mettl14* was also observed in other studies [38,39]. Knockdown of METTL14 in CD34^+^ HSPCs promotes monocytic differentiation, suggesting that under normal conditions it inhibits myelopoiesis [37]. METTL14 maintained HSC self-renewal and proliferation by directly targeting transcripts for the MYB and MYC transcription factors. *MYB* and *MYC* transcripts were decorated by m6A, which they lost upon METTL14 knockdown. This decreased the half-life of *MYB* and *MYC* mRNAs and reduced their binding to EIF3A, resulting in inefficient translation [37]. Lee et al. detailed the role of m6A in hematopoiesis even further [39]. The conditional knockout of Mettl3 in adult hematopoietic cells using poly I:C treatment of Mx1-Cre, Mettl3^fl/fl^ mice led to an early decrease in blood platelets and, at later time-points, a decrease in white blood cell counts. There was a significant drop in bone marrow cellularity and prominent splenomegaly, accompanied by an increase in HSCs in the spleen. Enlarged spleens with disorganized histological architecture, an accumulation of immature erythroblasts, and reduced B- and T-cell populations was also observed by others [40] in conditional Mettl3 knockouts. In the bone marrow, Lee et al. noted an early increase in the megakaryocyte progenitors Lin^−^/Sca-1^−^/c-Kit^+^/CD150^+^CD41^+^, as well as an expansion of the HSC and LSK cell pools in Mettl3^Δ/Δ^ animals 4 months after knockout [39]. The in vitro differentiation potential of single Mettl3 knockout HSC cells was defective with significantly fewer multi-potent granulocyte, erythrocyte, monocyte, and megakaryocyte (GEMM) colonies formed and a failure to differentiate into Mac1^+^/Gr-1^+^ myeloid cells. In contrast, Mettl3-deficient restricted progenitors formed colonies that were similar in number, size, and composition to wild-type cells, which suggested that in vitro the differentiation defect resides in HSCs and not progenitor cells. The competitive transplantation assays showed that Mettl3-deficient HSC cells were unable to reconstitute recipient mice. Following transplantation with Mx1-Cre, Mettl3^fl/fl^ bone marrow cells alongside wild-type competitors—but delaying knockout for 8 weeks to allow for engraftment in the recipient mice—the authors found that the reconstitution defects of Mettl3-deficient cells were mainly in the B-cell (B220^+^) and myeloid lineages (Mac1^+^). At 20 weeks after knockout, Mettl3-deficient HSC numbers in the bone marrow were similar to the controls, but there was a reduction in CMP, GMP, and MEP progenitors derived from Mettl3^Δ/Δ^ HSCs. This confirmed that in vivo HSC self-renewal is not significantly impacted by Mettl3 knockout, but their differentiation potential is [39]. Using *Lysm*-Cre Mettl3^fl/fl^ mice, in order to knockout Mettl3 specifically in myeloid cells, the authors showed that it is not required for myelopoiesis—the mice had normal peripheral blood cell counts, normal bone marrow and spleen cellularity, and normal frequencies of myeloid cells in the bone marrow. In addition, Mettl3-deficient bone marrow myeloid cells from these mice were capable of differentiating into macrophages with normal morphology and function. Methylated RNA immunoprecipitation sequencing (MeRIP-seq) on wild-type HSCs revealed m6A-target transcripts with significant enrichment of genes important for hematopoiesis, including *Myc*, *JunB,* and *Tet2*. Mettl3-deficient HSCs failed to upregulate Myc when prompted to differentiate, and ectopic expression of Myc protein could rescue the differentiation defects in vitro. This suggests that Mettl3-mediated m6A modification of *Myc* transcripts is required for normal HSC differentiation to proceed [39]. The role of m6A modification of Myc during HSC lineage commitment was further dissected by Cheng et al. [40], who observed that 3 weeks after conditional Mettl3 knockout (Mx1-Cre, Mettl3^fl/fl^), in the bone marrow, there was an increase in cells that phenotypically resemble HSC. An increase was also noted for the LSK cells, for MPP2 (LSK, CD150^+^CD48^+^) progenitors—biased toward megakaryocyte and erythroid lineages—and for the MPP4 cell population (LSK, CD150^−^CD48^+^), which is biased toward lymphoid lineages. At the same time there was a significant reduction in CMP (Lin^−^Sca-1^−^c-Kit^+^CD34^+^FcrR^−^) and GMP populations (Lin^−^Sca-1^−^c-Kit^+^CD34^+^FcrR^+^), confirming the previous observation that the knockout of Mettl3 disrupts the differentiation of HSC to myeloid progenitor cells. Indeed, in transplantation studies, Mettl3-knockout total bone marrow cells inefficiently reconstituted HSC, progenitor, and mature cell compartments (granulocytes, megakaryocytes, B- and T-cells). Interestingly, Mettl3-deficient HSCs lose their quiescent phenotype, exit the G_o_ stage of the cell cycle, and exhibit increased metabolic activity; signs that they have lost their HSC identity. Three weeks after deletion of Mettl3, a single cell RNA-sequencing analysis of Mettl3-deficient LK cells (Lin^−^/c-Kit^+^) revealed the emergence of two HSC-like intermediate cell populations, which are incapable of normal commitment to multipotent progenitors. The first showed reduced self-renewal and the second was primed toward the megakaryocyte fate. The gene expression signatures of Mettl3 conditional knockout HSCs confirmed the depletion of self-renewal genes and demonstrated an enrichment of ribosome and protein translation-related gene-sets, which are normally characteristic of MPPs and not HSC cells. The analysis for direct Mettl3 and m6A targets revealed a small number of potential transcripts: *Myc*, *Fmnl1*, *Lmo2*, *Ankrd13a*, *Zyx*, *Gnas*, *Son*, and *Zc3h4* [40]. Wild-type HSCs can undergo either symmetric self-renewal, symmetric lineage commitment, or asymmetric cell divisions. Cheng et al. discovered that Mettl3-knockout HSCs were less able to undergo symmetric commitment than wild-type cells. *Myc* expression and mRNA half-life were reduced in Mettl3-knockout HSCs. The overexpression of Mettl3, but not of catalytically dead mutant Mettl3^mut^, could rescue Myc expression—and, to a certain extent, the symmetric commitment—and could enhance the engraftment of Mettl3 knockout HSCs in transplantation experiments. Collectively, these data suggest that the symmetric lineage commitment of HSCs is regulated by m6A through the control of the stability and level of *Myc* mRNA [40].

### 3.3. Hematopoiesis during Infection and a Possible Role for m6A

Hematopoiesis during infection aims to preserve and maintain the balance in bone marrow niches and the functionality of HSCs. This supports quick and adequate lineage commitment and differentiation along one or more specific progenitor paths. HSCs can respond directly or indirectly to signals derived by pathogens or via growth factors, pro-inflammatory cytokines, and chemokines. Thus, during infection an emergency state arises in the bone marrow niche, where the generation of CMPs, myelopoiesis and granulopoiesis is enhanced, and HCSs are adapted to regulate this new emergency condition by the secretion of various paracrine factors [41]. Correspondingly, the genes involved in myeloid activation have a more stable m6A stoichiometry [11], and the expression of *Mettl3* and *Mettl14*, as well as m6A, decrease progressively during myeloid differentiation [38,39]. This suggests that myelopoiesis might be a more favorable path during infection and less prone to changes in m6A modification. Indeed, granulocytes, which are key for the early response to pathogens and possess a very sensitive regulation of granulopoiesis, exhibit a lower expression of *Mettl14* [37] compared to other cell types.

### 3.4. M6A in Innate Immune Cell Function

**Figure 3:**
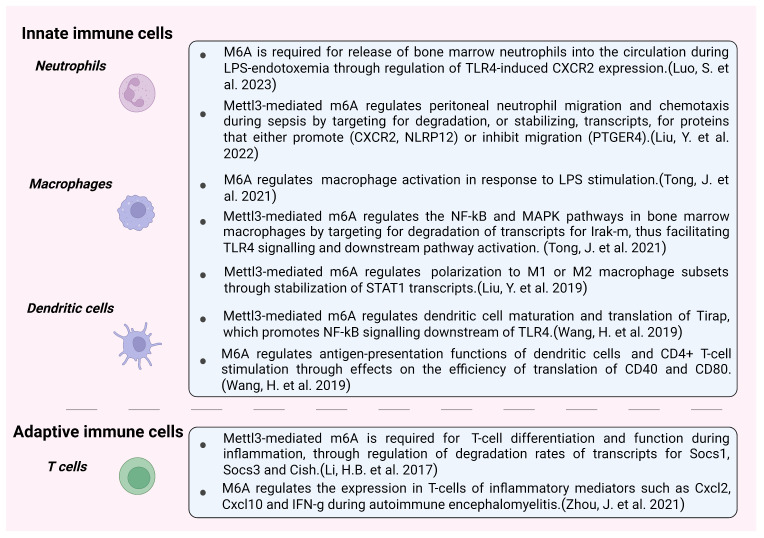
Significance of Mettl3-mediated m6A for regulation of innate and adaptive immune cells. Figure created with BioRender.com. Summaries of the results from previously published investigations into the role of m6A for the regulation of differentiation from naïve to effector immune cells, for immune cell response to stimulation, and for control of intrinsic cell-type-specific functions. Detailed discussions of the referenced studies can be found in the main text [42,43,44,45,46,47,48].

Innate immune cells (neutrophils, macrophages, and dendritic cells) are essential for the host response to invading pathogens, and neutrophils are the most abundant leukocyte among them. They are recruited to sites of inflammation or infection, driving initial inflammation and immune responses, and carry out their cell-type-specific functions (e.g., NETosis and phagocytosis). They secrete chemokines, which attract additional cell types and also play a role in the resolution of the inflammatory insult and clearance of the infection, mitigating tissue damage and/or initiating repair by the secretion of cytokines and growth factors. The role of m6A in regulating innate immune cell activation, migration, secretion of inflammatory mediators, and other protective processes has come into focus (Figure 3).

Recently, it was shown that Mettl3 is important for the mobilization of bone marrow neutrophils in mice with LPS-induced endotoxemia through modulation of the expression of the chemokine receptor CXCR2 [42]. Luo et al. have also demonstrated that activation of the TLR4 pathway is compromised in Mettl3-deficient bone marrow neutrophils isolated from *Lysm*-Cre, Mettl3^fl/fl^ mice [42]. M6A modification of the mRNA for *TLR4* stabilizes the transcript, promotes its translation, and increases TLR4 expression levels. This contributes to elevated cytokine secretion and enhanced TLR4 signaling via adaptor protein MyD88, as well as the downstream activation of the transcription factor NF-kB signaling pathway [42]. Indeed, it was suggested that METTL3 knockdown leads to increased expression of the splice variant of MyD88–MyD88S, which inhibits the inflammatory cytokine production in an LPS-induced inflammatory response instead of stimulating it [49]. Liu et al. reported that human neutrophils, both primary and differentiated HL-60 cell cultures, downregulate the m6A demethylase ALKBH5 in response to *E. coli* infection, with a corresponding increase in global m6A levels [43]. Intriguingly, the previously published gene expression datasets [50] [GSE6535] show that leukocytes isolated from critically ill patients with Gram-negative sepsis exhibit significantly reduced *ALKBH5* expression. This suggests that Alkbh5 is involved in the host defense against bacterial infection. Using cecal ligation and puncture (CLP), a mouse model of a bacterial infection resembling clinical sepsis, Liu et al. confirmed that both blood and peritoneal neutrophils from septic mice have reduced Alkbh5 levels [43]. An increased bacterial load was detected in peritoneal lavage samples and in the blood from Alkbh5-knockout mice with CLP compared to wild-type mice. This was also observed in the spleen, lung, liver, and kidney, pointing to an exacerbated systemic bacterial infection in the absence of the m6A demethylase. CLP generally results in efficient recruitment of neutrophils to the peritoneal cavity. However, in Alkbh5-deficient mice, the number of peritoneal neutrophils and, to some extent, macrophages is significantly decreased. This was mechanistically explained by the reduced cell surface expression of the receptor Cxcr2 in Alkbh5-knockout neutrophils from the blood and bone marrow. Collectively, this revealed a mobilization defect in Alkbh5-deficient neutrophils and a reduced ability to recruit these cells to the sites of infection. RNA-sequencing analysis of peritoneal neutrophils identified differentially expressed genes in wild-type and Alkbh5-knockout cells from early- (12 h) or late-stage CLP-induced sepsis (36 h). At both time-points, the gene ontology analysis revealed *neutrophil migration* and *neutrophil chemotaxis* as the most significantly affected processes. Alkbh5-knockout peritoneal neutrophils were shown to downregulate genes that promote migration (e.g., NLR family pyrin domain containing 12; *Nlrp12*) and upregulate genes with inhibitory functions in neutrophil migration (e.g., prostaglandin E receptor 4; *Ptger4*). The M6A-sequencing analysis of human dHL-60 cells revealed the enrichment of m6A on transcripts for these and other migration-related differentially expressed genes, which was increased in ALKBH5-knockout cells. This affected the rate of degradation of the transcripts, with a decreased stability of transcripts for *CXCR2* and *NLRP12* and increased stability of transcripts for *PTGER4*, tenascin C *(TNC*), and WNK lysine deficient protein kinase 1 *(WNK1*) compared to wild-type cells. Collectively, this study proposes a positive role of Alkbh5 in the innate defense against bacterial infection through regulation of intrinsic neutrophil properties, such as polarization in response to stimuli, chemotaxis, and migration [43].

Macrophages are also a major player in the host response to invading pathogens. Using a pooled CRISPR-Cas9 screen to identify RNA binding proteins important for macrophage activation, Tong et al. revealed that components of the m6A methyltransferase complex (*Mettl3*, *Mettl14*, and *Rbm15*) are required for this process [44]. Bone marrow-derived macrophages (BMDMs) from mice in which Mettl3 was knocked out in the myeloid lineage (*Lysm*-Cre, Mettl3^fl/fl^) showed a reduced capacity to respond to LPS stimulation, as assessed by the decreased expression of *Tnf-α*, *IL-1β*, *IL-6*, and *IL-12* compared to wild-type cells. siRNA-mediated knockdown of the reader protein Ythdf1 also resulted in decreased *Tnf-α* expression after LPS, and the effect was cumulative when Ythdf1/2/3 were knocked down simultaneously. The KEGG enrichment analysis of differentially expressed gene-sets from the RNA-sequencing of LPS-treated Mettl3 knockout Raw 264.7 macrophages revealed the *spliceosome-* and *viral infection-related* pathways as the most significantly affected. In addition to their defective macrophage activation phenotype in vitro, Mettl3^Δ/Δ^ mice were more susceptible to oral infection with *S. typhimurium* and showed an increased bacterial load in the cecum, spleen, and liver compared to infected wild-type controls. The LPS-treated Mettl3-knockout BMDMs exhibited a decreased capacity for activation of the NF-kB and MAPK pathways—as shown by the decreased phosphorylation of NF-kB p65, p38, and the Jnk and Erk kinases—which are essential to mount an effective immune response. Tong et al. explained this observation with the increased mRNA and protein expression levels of Irak-m, a negative regulator of TLR4 signaling, in both unstimulated and LPS-treated Mettl3-knockout BMDMs. *Irak-m* was confirmed as a direct target of Mettl3, with m6A modified *Irak-m* transcripts from wild-type BMDMs being degraded more quickly compared to transcripts that lose methylation as a result of Mettl3 knockout [44]. Collectively, this study suggests that m6A is important for macrophage activation and the expression of pro-inflammatory cytokines and functions as a positive regulator of macrophage antibacterial properties in vivo. In macrophages, Mettl3, through its m6A-mediated downregulation of Irak-m expression, facilitates TLR4 signaling and the activation of downstream pathways, which enhance the expression of cytokines, such as *Tnf-α* [44]. Alternatively, METTL3 can mediate the polarization of macrophages to the M1 subset via m6A modification and stabilization of signal transducer and activator of transcription 1 *(STAT1*) mRNA and elevated expression of the STAT1 protein, which regulates IL-6, IFN-γ production, and cell-mediated immunity. By contrast, the knockdown of METTL3 using siRNA encouraged M2 polarization that may promote the anti-inflammatory phenotype and tissue repair [45].

A similar role for Mettl3 and m6A in facilitating cellular activation was also reported for dendritic cells by Wang et al. [46] The authors demonstrated that the maturation of bone marrow dendritic cells along the trajectory immature BMDC>LPS-treated mature BMDC>regulatory DCs is associated with changes in m6A levels; m6A being highest in mature DCs (m6A/A = ~0.6%), as measured using quantitative mass spectrometry. This was accompanied by increased Mettl3, Mettl14, and Wtap expression, suggesting that m6A is required for DC maturation. The gene ontology analysis of gene-sets with m6A peaks in mature DCs compared to the other DC types showed enrichment for cell cycle and immune system processes. The KEGG pathway analysis revealed *metabolic-* and *infection-related* pathways as significantly enriched. In particular, gained m6A peaks in mature DCs were enriched for transcripts of genes belonging to Nod-like receptor, TNF, and NF-kB signaling pathways, suggesting a role for m6A in DC cell activation and function. Indeed, following LPS stimulation, splenic- and bone-marrow-derived dendritic cells from Mettl3 conditional knockout mice (*CD11c*-Cre, Mettl3^fl/fl^) had decreased expression of MHC class II (I-A^b^); the co-stimulatory molecules CD40, CD80, and CD86; and the cytokines Tnf-α, IL-6, and IL-12p70, indicating decreased antigen-presenting functions. In vitro, Mettl3 knockout mature BMDCs were defective in stimulating the proliferation and Ifn-γ production by CD4^+^ T-cells. This was also true in vivo, where the abundance of labeled OT-II CD4^+^ T-cells and their Ifn-γ expression in the popliteal lymph nodes of mice, immunized with Mettl3 knockout DC cells and pulsed with ovalbumin peptide (323–339), was decreased compared to wild-type controls. These defects could be rescued by overexpression of wild-type, but not catalytically inactive, Mettl3 in the dendritic cells. This suggests that m6A modification in dendritic cells is required for them to be able to stimulate CD4^+^ T-cells. The RNA-sequencing analysis of LPS-treated mature Mettl3 knockout BMDCs confirmed the downregulation of MHC class II *H2-Eb2* and cytokines *IL-6* and *IL-12b*, which was not due to the increased degradation of these transcripts. Rather, following LPS stimulation, the Mettl3-knockout BMDCs had significantly reduced Tirap protein expression, as well as decreased phosphorylation of the signaling molecules Tak1, Ikkα/β, Erk, Jnk, and NF-kB p65, compared to wild-type DCs. This points to a defective activation of TLR4/NF-kB and MAPK pathways in response to LPS. *Tirap*, *Cd40,* and *Cd80* transcripts were shown to be modified by m6A, the level of which was reduced in Mettl3-deficient mature DCs. This resulted in a decline in the efficiency with which they were translated. Collectively, this study demonstrates that m6A modification is required for DC maturation and efficient translation of Tirap, which promotes NF-kB signaling downstream of TLR4 in response to external stimuli. M6A is also required for the efficient translation of the co-stimulatory proteins CD40 and CD80, which are necessary for the antigen-presentation functions of DCs and CD4^+^ T-cell proliferation and stimulation [46].

### 3.5. M6A in Adaptive Immune Cells

Adaptive immune cell homeostasis and function is also controlled, at least in part, by m6A modifiers. M6A modification is a key regulator of T-lymphocyte development in the thymus, where naïve T-cells acquire specific T-cell receptors (TCR) and mature to CD4 or CD8 T-cells; also in the periphery, where mature naïve T-cells encounter the antigen/MHC complex at antigen-presenting cells, and polarize and differentiate to various CD4^+^ helper cell populations (i.e., T_H_1, T_H_2, T_H_ regulatory cells, and T_H_17) or directly kill the infected cells (CD8^+^ T-cells). A recent study showed that Wtap-dependent m6A modification regulates the differentiation of thymocytes and their survival, as well as T-cell activation-through the modulation of intracellular calcium mobilization—following the engagement of the TCR and expansion of specific T-cell clones [51]. Indeed, the same study demonstrated that Wtap controls the generation of a rare population of regulatory T-cells in the thymus, which is important to sustain peripheral T-cell tolerance [51].

Recently, Li et al. demonstrated that Mettl3-deficient naïve T-cells are unable to undergo proper differentiation into functional effector T-cell populations [47]. The authors reported that the conditional knockout of Mettl3 in T-cells (*CD4*-Cre, Mettl3^fl/fl^) resulted in an increase in the number of naïve CD4^+^ T-cells in the spleen and in the mesenteric and peripheral lymph nodes of knockout mice. At the same time, there was a reduction in T_H_1 and T_H_17 cells concomitant with increased T_H_2 compared to wild-type animals. The Mettl3-deficient naïve T-cells were defective in adoptive transfer colitis experiments using Rag2-knockout mice, which cannot produce mature T- and B-cells, and were unable to promote the development of colitis, in contrast to wild-type T-cells [47]. No immune cell infiltration and colonic inflammation could be detected in recipients of Mettl3-knockout T-cells, as these cells exhibited significantly slower proliferation and mostly remained restricted to the lymph nodes. A similar result—mild colitis and less lymphocyte infiltration in the colon—was observed by Zhou et al. using Alkbh5-knockout CD4^+^ T-cells [48]. Interestingly, Mettl3-knockout T-cells maintained their naïve marker CD45RB^high^ and could not differentiate into effector T-cells (CD45RB^low^) [47]. Mechanistically, this differentiation defect was explained by the inability to activate the Jak/Stat signaling pathway, as evidenced by the decreased phosphorylation of Jak1 and Stat5 following the stimulation of Mettl3-deficient T-cells with IL-7 [47]. These cells also had elevated basal phosphorylation levels of Erk and Akt even prior to interleukin treatment, which might explain their viability up to 10 weeks after transfer. The RNA sequencing of naïve CD4^+^CD25^−^ CD45RB^high^ cells from splenic and lymph node T-cell populations revealed that the expression levels of *Socs1*, *Socs3,* and *Cish*—genes important for the inhibition of the response to cytokines through the Jak/Stat signaling pathway—were increased in Mettl3-deficient T-cells. The KEGG analysis of upregulated pathways revealed *cytokine–cytokine receptor* interactions and the Jak/Stat pathway. The upregulation of *Socs1/3* and *Cish* was due to slower degradation rates of the respective transcripts following their loss of the m6A modification. Collectively, this study suggests that m6A can control naïve T-cell proliferation and differentiation in response to stimuli by decorating the transcripts of inhibitory molecules, such as Socs, which leads to their quicker degradation and permits Jak1/Stat5-mediated signaling downstream of IL-7 to proceed [47]. A similar effect of m6A-loss on IL-2/Stat5 in regulatory T-cells was also demonstrated [52]. Tong et al. reported that the conditional knockout of Mettl3 in regulatory T-cells (*Foxp3*-Cre, Mettl3^fl/fl^) results in severe autoimmune disease, accompanied by the increased percentage of IL-17 and Ifn-γ producing CD4^+^ cells, indicating a defect in T_reg_ suppressive function. There was a similar upregulation of *Socs1/2/3*, *Cish,* and *Asb2* genes in Mettl3-deficient T_reg_ cells, which inhibited IL-2/Stat5 signaling [52].

Expression levels of the m6A demethylase, *Alkbh5*, modestly increase in differentiated T_H_1, T_H_2, and T_H_17 compared to naïve T-cells, suggesting that it may also be important for effector T-cell functions [48]. Using experimental autoimmune encephalomyelitis (EAE) as a model of autoimmune disease, Zhou et al. demonstrated that mice with Alkbh5 knockout specific to T-cells (*CD4*-Cre, Alkbh5^fl/fl^) exhibit ~2-fold reduction in lymphocyte infiltration and less spinal cord demyelination compared to wild-type mice and, overall, were resistant to the induction of EAE using immunization with myelin oligodendrocyte glycoprotein peptide (MOG_35–55_) [48]. Alkbh5-knockout T-cells produced less Ifn-γ in the CNS, suggesting a protective effect against neuroinflammation in this disease model. In addition, fewer neutrophils were recruited to the CNS in *CD4*-Cre, Alkbh5^fl/fl^ mice compared to wild-type controls with EAE. The pathway enrichment analysis of RNA-sequencing data from CNS-derived CD4^+^ T-cells isolated from *CD4*-Cre, Alkbh5^fl/fl^ and control mice with EAE showed that the *IL-17, TGF-β,* and *TNF* signaling pathways were significantly downregulated in Alkbh5 knockout cells. There was reduced expression of *Cxcl2*, *Cxcl10*, and *Ifn-γ* in Alkbh5 knockout CD4^+^ T-cells. Moreover, *Cxcl2* and *Ifn-γ* transcripts were shown to be direct targets for m6A modification, which increased significantly upon Alkbh5 knockout. Increased *Cxcl2* and *Ifn-γ* m6A modification led to decreased transcript stability and resulted in reduced protein expression [48]. The results of this study collectively suggest a disease-modulating role for Alkbh5 during autoimmune encephalomyelitis through effects on the stability of transcripts for inflammatory mediators in T-cells isolated from the CNS.

## 4. RNA Modification as a Biomarker for Host–Pathogen Interactions

### 4.1. M6A Effects on the Viral Life Cycle

The host cell’s METTL3/METTL14 and associated complex can place m6A on genomes and transcripts of diverse viruses, with effects on their life cycle, virulence, and pathogenesis. Viruses belonging to *Flaviviridae* [53,54], *Orthomyxoviridae* [55], *Coronaviridae* [56], *Pneumoviridae* [57], *Retroviridae* [58,59], *Hepadnaviridae* [60], and *Herpesviridae* [61,62] families possess RNA decorated by m6A, which can negatively or positively regulate infection, depending on the species (Table 1).

These effects are mediated through diverse mechanisms. For example, Hepatitis C virus replication and infectious particle production increase following the siRNA-mediated depletion of both METTL3 and METTL14, revealing these m6A writer proteins as negative regulators of HCV infection [53]. The same effect was observed following the depletion of the m6A readers YTHDF1-3. Mutational inactivation of the HCV genome—within the viral E1 gene—at four m6A sites that can be bound by YTH proteins resulted in a 3-fold increase in viral titers. This effect was not mediated through enhanced HCV RNA replication but via an increase in viral particle assembly and a preferred interaction of the HCV core protein with the unmodified viral RNA genomes, which facilitates packaging of the virus [53]. In the Zika virus (strain MR766), m6A was quantified by Lichinchi et al. using LC-MS/MS and shown to be surprisingly high (m6A/A = 3%), with MeRIP-sequencing revealing 12 major m6A peaks spanning the ZIKV genome [54]. Half of them cluster in the NS5 region and within the 3′UTR. This distribution pattern was also seen by Gokhale et al. [53] for the DAK and PR2015 strains of ZIKV and appears to be conserved among other flaviviruses such as Dengue, Yellow fever, and West Nile virus. It was also similar to the pattern observed in the HCV genome, where m6A is enriched in the NS5B region [53]. The depletion of METTL3 and METTL14 by shRNA in 293T cells leads to increased ZIKV titers and RNA levels, while their overexpression had the opposite effect [54]. The silencing of YTHDF1-3 by RNAi increased ZIKV replication, with YTHDF2 having the strongest effect. Thus, m6A and direct binding of the Zika virus RNA by the YTHDF1-3 reader proteins appear to be a negative regulator of the virus, similar to the HCV example above; however, the exact mechanism of their effect on ZIKV replication remains obscure [54].

The human metapneumovirus (HMPV) genome, antigenome, and viral transcripts all contain m6A peaks, as shown by Lu et al. using an immunoprecipitation-based approach (m6A-seq) [57]. On both the negative and positive RNA strands, m6A is enriched in the G-region of the genome. In contrast to the flaviviruses HCV and ZIKV, the m6A modification of HMPV viral RNA has a positive effect on viral replication. The overexpression of METTL3 and METTL14, as well as the YTH-family of proteins, led to a significant increase in viral titers and G- and N-protein expression [57]. By mutating m6A sites within the G-region, Lu et al. created recombinant viruses with reduced m6A levels [57]. Infection with these m6A-deficient viruses led to the inhibition of viral replication and the production of fewer N- and G-proteins. They also formed smaller spots in immunostaining plaque assays and developed cytopathic effects in A549 cells earlier than the regular HMPV. m6A-deficient viruses induced a significantly increased Type I IFN response both in vitro and in vivo. This effect was most pronounced using a recombinant virus lacking all 14 potentially available m6A sites in the G-region and was completely lost in RIG-I or MAVS-knockout A549 cells, suggesting that it is mediated through the RIG-I pathway. Interestingly, infection with m6A-deficient viruses also significantly increased *RIG-I* expression levels, especially in the early post-infection period (8–16 h) [57]. Viral infection is known to activate the RIG-I pathway and induce downstream Type I IFN production; however, the m6A modification of HBV, HCV (Kim et al.) [63], SARS-CoV-2 (Li et al.) [56], and HMPV RNA (Lu et al.) [57] in the region recognized by this receptor can inhibit the interaction with RIG-I and dampen the innate immune response to these pathogens. In the case of hepatitis B and C viruses, Kim et al. observed that the depletion of METTL3 and METTL14 by siRNA led to an increase in IRF3 phosphorylation and a downstream increase in IFN-β expression levels [63]. The authors found that when the HBV pre-genomic RNA (pgRNA) and the HCV RNA genome were modified by m6A—within or close to their pathogen-associated molecular pattern regions (PAMP)—they are bound by YTHDF2 and -DF3 proteins, which negatively impacts RIG-I recognition and pathway activation, as well as the overall antiviral response [63]. This can be thought of as a protective mechanism used by these viruses in order to escape the host innate immune system. A similar strategy of immune evasion is also used by SARS-CoV-2. Using LC-MS/MS, Li et al. estimated that this virus contains ~8 m6A modified sites (m6A/A = 0.096%), enriched in the nucleocapsid regions of the genome (N-3 and N-4) [56]. In Caco-2 cells infected with USA-WA1/2020 SARS-CoV-2 virus, the knockdown of METTL3 or METTL14 by shRNA resulted in reduced viral N- and E-gene expression levels. The loss of m6A on viral RNA, as a result of METTL3 knockdown, led to the increased binding of RIG-I to it [56]. Similarly, introducing mutations at m6A sites that fall within DRACH motifs in the SARS-CoV-2 N-3 and N-4 regions led to an increased expression of inflammatory genes in Caco-2 cells and enhanced RIG-I binding to the mutant compared to the wild-type RNA [56]. In the case of HMPV, Lu et al. also found that RIG-I binds more efficiently to antigenome RNA from m6A-deficient viruses, triggering both IRF3 phosphorylation with downstream IFN induction and activation of the NF-kB pathway and cytokine production [57]. In vivo, m6A-deficient HMPV trigger an increased Type I IFN response, as assessed by IFN-β bioactivity in broncho-alveolar lavage fluid (BAL) samples from infected cotton rats when compared to mock infected animals or infections with non-mutant viruses. Reduced nasal replication and lung viral titers, as well as fewer lung lesions, were also observed, suggesting that m6A-deficient HMPV are less pathogenic [57].

Courtney et al. found that influenza A virus (IAV) replication also depends on the m6A modification of viral transcripts, which is beneficial for the virus [55]. The knockout of METTL3 in A549 cells led to decreased NP, NS1, and M2 viral protein expression up to 72 h post-infection with the IAV-PR8 virus, in addition to significantly reduced infectious viral particle production. The opposite effect was observed following the overexpression of the YTHDF2 reader protein. Using photo-crosslinking-assisted m6A-sequencing (PA-m6A-seq) of infected 293T cells, Courtney et al. demonstrated that this modification is present at multiple locations on viral mRNAs encoding HA, NP, NA, and M proteins, with corresponding YTHDF binding sites [55]. Multiple m6A-modified positions on the negative strand vRNA were also detected. Importantly, IAV-PR8, in which essential sites for m6A methylation of the HA segment were mutated and, thus, rendered unmodifiable, either on the mRNA (8 sites) or the vRNA (9 sites), had decreased HA mRNA and protein levels and were less pathogenic in vivo. However, why this occurs remains unclear [55].

The knockdown of METTL3 or METTL14 using shRNA in MT4 cells and the subsequent infection with the HIV-1 virus (clone LAI) led to a significant decrease in viral replication, as assessed by gp120 mRNA expression and p24 capsid protein levels [58]. This effect was cumulative in double METTL3/14-depleted cells. Depleting the m6A demethylase, ALKBH5, caused the opposite effect. Using MeRIP-sequencing, Lichinchi et al. [58] identified 14 methylation peaks spread across the viral genome, located in coding and non-coding sequences, splicing junctions, and splicing enhancers/silencers. Topologically, these peaks were highly enriched within the *vif* and *vpr* coding sequence; the splicing regulatory regions ESEVpr, ESS2p, ESS2, and ESS3a; the Rev response element (RRE); and also within the *env*, *rev,* and *nef* coding sequences. The presence of some of these major m6A peaks within the viral genome, specifically the 3′-localized, was also confirmed by Kennedy et al. [59] using PA-m6A-sequencing of infected CD4^+^ CEM-SS T-cells. The binding of the viral Rev protein to the RRE RNA element within the *env* gene is a necessary step in viral replication, which leads to the formation of nuclear export complexes and facilitates the transport of viral RNA into the cytoplasm. Lichinchi et al. found that METTL3/14 knockdown decreased m6A methylation at two conserved adenosines within the RRE hairpin structure (A7877 and A7883) [58]. This negatively impacted its interaction with Rev, severely reduced the export of viral RNA from the nucleus, and inhibited viral replication. Conversely, ALKBH5 knockdown enhanced the formation of Rev–RRE complexes and, consequently, nuclear export and viral replication [58]. Kennedy et al. also concluded that m6A acts as a positive regulator of HIV-1 replication [59]. They showed that YTHDF1-3 proteins predominantly bind to four clusters of m6A-modified sites corresponding to *env*/*rev*, *nef,* and the LTR R and NF-kB-binding (U3) regions of the HIV-1 genome. These clusters were mostly conserved between different HIV-1 viruses (NL4-3, BaL, JR-CSF). In the infected 293T cells, overexpression of the YTHDF1-3 proteins enhanced the expression of *Nef*, *Tat,* and *Rev* mRNA and the viral genomic gRNA, with YTHDF2 having the strongest effect. These results were also reproduced in YTHDF2 overexpressing CEM-SS T-cells.

Collectively, these examples illustrate different viral replication and immune system evasion strategies mediated through the m6A RNA modification pathway.

### 4.2. M6A Effects on the Host Response to Viral Infection

The discovery that internal RNA modification on host transcripts can shape the host antiviral response has generated much interest. For example, Li et al. demonstrated that in Caco-2 cells infected with USA-WA1/2020 SARS-CoV-2, the knockdown of METTL3 led to downregulation of proviral host genes, such as *ACE2*, in addition to *B4GALT7*, *NDST1,* and *TMPRSS4*, which also lose m6A (as assessed by MeRIP-seq) [56]. This suggests a role for METTL3 in the host’s response to the virus. In both METTL3- and METTL14-depleted Caco-2 cells, RNA-sequencing and qRT-PCR revealed upregulated immune response genes following infection (including IL-6, IL-8, CCL20, CXCL1, and CXCL3), while IFN and interferon-stimulated genes (ISGs) remained largely unaffected. Similarly, the METTL3 knockdown in a different cell line, Calu-3, significantly increased inflammatory cytokine/chemokine gene expression and also expression of IFN-β, STAT1, STAT2, and IRF7. Collectively, this suggests that METTL3 depletion leads to the induction of inflammatory genes in response to infection with SARS-CoV-2 that is mostly independent of IFN-induction, irrespective of the cell type. Mechanistically this is explained by the activation of the NF-kB pathway through enhanced phosphorylation of IkBα and IRF3 in infected METTL3-depleted cells. In 2020, Blanco-Melo et al. [67] reported results from RNA-sequencing using lung tissue from deceased COVID-19 patients and observed little Type I (IFN-β) or Type III (IFN-λ) response to SARS-CoV-2 compared to lung biopsy samples from healthy individuals. This was accompanied by an increased expression of genes involved in the innate or humoral responses; a phenomenon the authors referred to as an *imbalanced host response*. Some of these, such as IL-6, CCL8, CXCL9, and CXCL16 were also confirmed in serum samples from patients. Interestingly, when Li et al. reanalyzed this dataset, they observed a significant downregulation of METTL3 and METTL14 expression, coincident with increased inflammatory gene expression [56]. The argument that decreased METTL3 levels might be associated with the most severe COVID-19 cases was also strengthened by reanalysis of single cell RNA-sequencing data of BAL epithelial cells from patients stratified by disease burden (moderate vs. severe disease) [68].

The changes in RNA modification levels that occur on host transcripts following infection are beginning to be characterized. One potential role of m6A in this context comes from Winkler et al. [65] who showed that this modification acts as a negative regulator of the expression of IFN-α, IFN-β, and a number of ISGs. They observed that following the knockdown of METTL3 or YTHDF2, infection leads, through effects independent from the viral mechanisms, to the upregulation of many genes involved in the Type I IFN response, ultimately restricting viral proliferation. Underlying these non-viral mechanisms are changes to interferon transcript stability, with m6A-modified *IFNA* and *IFNB* transcripts being degraded faster. In contrast, these transcripts were stabilized when METTL3 and YTHDF2 were knocked down in the cells. Importantly, this appeared to be a conserved mechanism observed following infection with diverse viruses, such as HCMV, IAV, Adenovirus, and vesicular stomatitis virus (VSV). In all cases, the infection of METTL3- and YTHDF2-depleted cells led to enhanced *IFNB* and *ISG15* expression [65]. The Type I IFN response and downstream induction of many ISGs is necessary to mount an effective antiviral defense. In order to further understand the post-transcriptional regulation of ISG expression, McFadden et al. [69] investigated the m6A modification that occurs specifically on transcripts of genes induced by IFN-β and found that many ISGs are modified. Using the MeRIP-seq of IFN-β treated Huh7 cells, the authors observed that m6A modification across the induced ISG transcripts is wide-spread. Indeed, ~85% of the ISGs upregulated >4-fold following the interferon treatment were modified. Following the IFN-β treatment of cell lines, in which METTL3 and METTL14 were depleted using siRNA, certain ISG gene products (IFITM1 and MX1) were only weakly upregulated when compared to control cells. Conversely, IFN-β treatment of cells overexpressing both METTL3 and METTL14 had the opposite effect—it resulted in a higher induction of some ISGs in response to IFN-β. The reduced IFITM1 and MX1 protein levels in METTL3/14-depleted cells was explained by less of their transcripts binding to 80S ribosomes and a shift from the heavy to the light polysomal fraction, resulting in impaired translation. At the same time, YTHDF1 binding to *IFITM1* mRNA in an m6A-dependent manner was necessary and sufficient to increase its translation in response to IFN-β. However, it should be noted that a general effect of METTL3/14 depletion resulting in decreased ribosomal occupancy was observed for most, but not all, m6A-modified ISG transcripts.

The redistribution of m6A within host transcripts in response to a viral infection was also reported [54,58]. In MT4 cells infected with HIV-1 LAI, Lichinchi et al. [58] found that while the infection itself does not affect overall m6A topologies, host transcripts that are specifically methylated in response to the infection have more abundant methylation in the 5′UTR and CDS regions and less at introns and 3′UTRs compared to the distribution profiles of m6A observed in the total cellular mRNA. M6A-sequencing by Lichinchi et al. [54] showed that the infection of 293T cells with Zika virus also led to a net shift away from the 3′UTR and towards an accumulation at the 5′UTR of host transcripts. Of note, this was not confirmed in a later study by Gokhale et al. using a different cell type—Huh7 [64]. The analysis of gained/lost peaks by Lichinchi et al. showed that, following the infection, newly acquired m6A enrichment peaks were preferentially deposited at CDS and exon junctions and, to a lower extent, at 3′UTRs. In contrast, peaks were mostly lost from exon junctions and 3′UTRs and, to a lesser degree, from 5′UTRs [54]. This indicates that both HIV-1 and ZIKV infection can affect host mRNA splicing and alternative exon usage through m6A. Gokhale et al. provided a more detailed view of the specific cellular transcripts, which were affected by infection with four *Flaviviridae* family members: Dengue, West Nile, Zika, and the Hepatitis C virus [64]. While there was no sizable overall change in host cell %m6A/A in infected vs. uninfected cells (m6A/A = ~0.2%), there were m6A-modified gene transcripts that were differentially expressed in response to infection. The MeRIP-seq performed on RNA from infected Huh7 cells revealed common differentially expressed genes across all four viruses. Among them were upregulated genes belonging to the innate immune response (e.g., CXCL10, TNFAIP3, RELB) and ER-stress response pathways (e.g., ATF3, PPP1R15A, BBC3), two pathways known to be activated during a flavivirus infection. In addition, across the four viruses, there were 51 host genes that shared infection-induced changes to m6A peaks, mostly gained, that mapped to CDS and 3′UTR regions. Interestingly, these genes were not enriched for functional categories related to infection and included examples such as RIOK3, CIRBP, PER2, NOC2L, ALCAM, GOLGA3, and others. Using *RIOK3*—a transcript that gains m6A peaks in its 3′UTR—the authors showed that during infection with DENV, ZIKV, and HCV, this mRNA is modified in response to the activation of IRF3 and IFN-β and is consequently recognized and bound by YTHDF1, which promotes translation and leads to increased RIOK3 protein levels, even in the context of the global inhibition of cellular translation. This might be beneficial for DENV and ZIKV because independent overexpression of RIOK3 in infected Huh7 cells results in increased viral titers. For *CIRBP*—a transcript that loses m6A peaks—a different regulatory mechanism was observed. The activation of the ER-stress response during infection led to lower m6A modification of *CIRBP*, which resulted in alternative splicing of the transcript and reduced retention of an intron located downstream of the affected m6A peak. This led to the decreased expression of a longer isoform (CIRBP-L) containing the intron, which is inefficiently translated irrespective of infection status. The authors suggest that this might be a mechanism that allows for the consistent expression of the shorter isoform, CIRBP-S, during infection. Ectopic overexpression of both CIRBP-S and -L isoforms increased the infection by DENV, ZIKV and HCV, suggesting that CIRBP functions as a proviral gene in this context.

The changes to host m6A methylation that specifically occur in response to infection with HIV were reported. In the MT4 cells, infection with HIV-I LA1 led to ~30% increase in overall m6A modification of cellular mRNA [58]. In this study, 56 host cell transcripts were shown to be uniquely methylated upon infection, many of which were previously associated with HIV replication (MOGS, LEDGF, HNRNPK, TRAF2, HSPA1A, SRSF5, EIF3M, MBD2, and others). Many of the genes fall into gene ontology terms related to the viral infection and encode proteins with proviral functions, which are either required for or promote viral replication or are induced by the infection itself.

Collectively, these results show that m6A can suppress over-activation of the host interferon response to viral infection, can occur on specific host transcripts that are important for viral replication, and can potentially be redistributed internally in response to infection, thereby affecting splicing and translation efficiency.

## 5. Pharmacological Regulation of RNA Modifications—Implications for Infectious Diseases

Due to their extensive involvement in immune cell function and viral biology, modulating the activity of host regulatory proteins that set, read, or remove internal RNA modification can be used as a therapeutic option (Table 2).

Such an approach was recently reported by Burgess et al. [70] for the treatment of infection with β-coronaviruses. The authors used an RNAi screen to identify m6A modifiers (writers, erasers, or readers) that can affect viral replication in cell cultures infected with either the seasonal HCoV-OC43 or the pandemic SARS-CoV-2 virus. The depletion of the core methyltransferase components METTL3, METTL14, WTAP, as well as the reader proteins YTHDC1, YTHDF1, and YTHDF3 using siRNA could significantly decrease viral replication, as assessed by N-protein expression. When the authors used a highly-specific small molecule catalytic inhibitor of METTL3 [STM2457 [76]], they observed a 50% inhibition of viral replication for HCoV-OC43 at IC50 = ~21 µM and for SARS-CoV-2 at IC50 = ~17µM. The mechanism of viral inhibition by STM2457 was not through an enhanced Type I IFN host response, such as the increased expression of IFN-β or ISGs (IFIT2, ISG15, and OAS3). Rather, the observed antiviral properties of STM2457 were explained by the reduced accumulation of viral dsRNA in the infection-induced cytoplasmic vesicular structures (replication organelles), which function as sites of viral RNA replication. Treatment with the inhibitor resulted in reduced viral genomic and sub-genomic RNA species (g- and sgRNA) and consequently led to a broad defect in viral protein synthesis.

The antiviral effects of flavonoids against respiratory and non-respiratory viruses were investigated in depth using in vitro and in vivo studies [77]. Recently, a virtual screening carried out on natural products identified the flavonoids quercetin, luteolin, and scutellarin as METTL3 inhibitors [71]. These compounds show the same structure as the METTL3 degrader elvitegravir [78,79]. An in vitro evaluation confirmed their METTL3 inhibition potential, with IC50 values of 2.73, 6.23, and 19.93 μM, respectively. Of note, the pleiotropic activity shown by these polyphenolic compounds limits their use as chemical probes to study METTL3–METTL14 activity but offer the rational design for the development of new optimized derivatives.

Zannella et al. [72] showed that the small natural molecule Rhein (4,5-dihydroxyanthraquinone-2-carboxylic acid), an anthraquinone mainly isolated from medicinal plants [80], exerts a strong broad-spectrum reduction of infectivity caused by various coronaviruses, including SARS-CoV-2. The authors, by using the Vero E6 cell line, demonstrated that in vitro the replication of two common seasonal coronaviruses, i.e., HCoV-229E and OC43, and the pandemic SARS-CoV-2, could be inhibited by rhein through the regulation of the m6A pathway by selective inhibition of a m6A demethylase, FTO. Indeed, when cells were exposed at the same time to a virus and at the highest concentration of rhein (200 μg/mL), there was a two-fold increase in the RNA m6A level. The antiviral activity of rhein may also be ascribed to its ability to inhibit the angiotensin-converting enzyme 2 (ACE2) [81] and to activate multiple cytoprotective pathways [82].

The S-adenosyl-L-homocysteine (SAH) hydrolase inhibitor 3-deazaadenosine (DAA) is shown to inhibit m6A addition and to act as a broad antiviral inhibitor [73,75,83,84,85]. Bader et al. reported DAA to have an antiviral effect in the reproduction of the Rous sarcoma virus and the transformation of chick embryo cells (CEC), likely due to adenosyl-homocysteine hydrolase inhibition [73]. Similar results on RNA methylation were further obtained in vitro when CEC were infected with the influenza A virus [74]. The antiviral efficacy of DAA was tested against a wide variety of viruses not only using in vitro cell culture but also in small animal models, showing a reversible effect without apparent cytotoxicity. A proposed mechanism suggests that DAA blocking the cellular enzyme, S-adenosyl-L-homocysteine hydrolase, results in depletion of S-adenosyl-methionine (SAM), the methyl donor used by METTL3 to generate m6A [73]. Since DAA can inhibit all types of RNA methylation, it is difficult to conclude whether its antiviral effect comes from the inhibition of mRNA cap methylation or the internal m6A methylation [86,87]. An additional explanation for the antiviral efficacy of adenosine analogues may be ascribed to their anti-inflammatory effect on innate immune cells, mainly macrophages [87]. Thus, the in vivo administration of the inhibitor may prevent tissue damage mediated by activated macrophages and provide enough time for the host to mount a successful antiviral immune response.

Ye et al. demonstrated that Kaposi’s sarcoma-associated herpesvirus (KSHV) has evolved a mechanism to manipulate the host m6A machinery to its advantage in promoting lytic replication [66]. In this study, the authors observed an enhanced KSHV lytic gene expression when BCBL1 cells were treated with 1 μM meclofenamic acid (MA), a selective inhibitor of FTO [88], alone or in combination with a diester of phorbol for 24 h, while DAA blocking of m6A abolished the lytic gene expression and virion production.

In 2022, a UK-based biotechnological company, focused on the development of small molecule therapies targeting RNA modifying enzymes, announced a Phase I clinical trial (ClinicalTrials.gov identifier: NCT05584111) using STC-15—an orally bioavailable selective METTL3 inhibitor—for application in advanced solid tumors [89]. Compounds for clinical application in infectious diseases are also in the R&D pipeline. While the current focus is on oncology, other companies are also working to identify and validate compounds that target RNA-modifying proteins [90]. It should be kept in mind that the effect of m6A on viral titers and replication is not in the same direction for every viral species (Table 1). In addition, because, potentially, many host transcripts will be affected, such a therapeutic approach should be used cautiously and most likely for short durations only. As mentioned above, the genetic ablation of Mettl3 can seriously compromise the hematopoietic stem cell differentiation potential, especially into the myeloid and erythroid lineages. However, the effect of the acute depletion of Mettl3 in HSCs appears to be reversible [40]. Cheng et al. demonstrated that the transient knockdown of Mettl3 using siRNA led to an expected defect in cell engraftment in transplantation experiments in the short-term (4 weeks). This defect was mostly resolved by 12 weeks post-transplantation [40].

There are also other important points to consider: (1) how to target the therapy and consider direct vs. indirect effects.; (2) m6A affects all cell types in a context dependent manner—it may affect cellular differentiation, cell maturation, response to stimulation, and function; (3) m6A participates in essential processes (genomic stability, chromatin organization, transcriptional regulation, alternative splicing, translation efficiency, transcript stability), so inhibition of its writer proteins may potentially have a negative impact. While numerous studies have shed light on the physiological and pathological functions of RNA modifications during infection, we have seen a renewed interest in the pharmacological regulation of RNA modifications only in the recent SARS coronavirus outbreak. In vivo and clinical studies are still needed in order to fully harness the positive effects of synthetic and natural compounds targeting RNA-modifying proteins and develop effective therapies for infectious diseases. Analysis of the structure–activity relationship has shown that some compound scaffolds can be manipulated to overcome their clinical limitations and improve the antiviral activity. However, more efforts are required to determine the drug impact on overall RNA modification signatures, thus, avoiding the focus on particular modifications on specific transcripts.

## 6. Outlook

Functional studies continue to identify proteins with enzymatic activity involved in setting RNA modifications and RNA-binding proteins (RBPs) that can recognize them. The binding of RBPs results in specific outcomes depending on the cellular context (i.e., cell type, stem/progenitor cell vs. differentiated cell, resting vs. activated state). Despite major advances in RNA modification detection, significant gaps in our knowledge remain [9]. For example, we are just beginning to appreciate modification stoichiometry; however, most of what is known so far pertains to N6-methyladenosine. The development of single-nucleotide resolution approaches capable of capturing modification dynamics under different cellular conditions and treatments must continue for other modifications as well. Furthermore, not much if anything is known about the combinatorial RNA modification code, i.e., what effects do combinations of different modifications on a single transcript have on RNA metabolism and function. Recently, there has been a call for action to improve technologies for direct long-read sequencing of full-length RNA, as well as for better data analysis tools, which may eventually be used to comprehensively address this question [91]. Finally, most of the available RNA-modification literature is on bulk samples. In order to search for associations between phenotypic heterogeneity—in both in vivo and in vitro populations of the same cell type—and RNA modifications, as well as to investigate rare cell populations, the development of single cell approaches requiring low sample input continue to be needed [92,93].

Given the positive and negative effects of RNA methylation in distinct viral infections and the fact that its dysregulation may result in the emergence of a variety of diseases ranging from cancer to cardiovascular and neurological disorders, beyond contributing to viral infections, the discovery of highly selective new small molecule inhibitors or drugs targeting m6A “writers” or “erasers” may assist in furthering the understanding of the biological roles of these enzymes, in addition to contributing to the development of novel antiviral therapies.

## Figures and Tables

**Table 1:** The effect of viral and host m6A on pathogenesis and innate immune responses to infection.

Virus (Family)	Genome DNA/RNA	Effect on Viral Replication/ Pathogenesis	Effect on Innate/ IFN Response	Effect on Specific Genes/Signaling Cascades	Ref.
HCV (*Flaviviridae*)	(+)ssRNA	Neg[−]	METTL3/14 siRNA increases IFN-β expression.	M6A interferes with viral packaging and assembly. Negatively affects RIG-I recognition of viral RNA and pathway activation. Differentially expressed m6A-modified host transcripts for innate immune response and ER-stress response pathways.	[53,63,64]
Zika (*Flaviviridae*)	(+)ssRNA	Neg[−]	n/a	Differentially expressed m6A-modified host transcripts for innate immune response and ER-stress response pathways.	[54,64]
Influenza A (*Orthomyxoviridae*)	(−)ssRNA	Pos[+]	M6A is a negative regulator of IFN-α, IFN-β, and ISG expression. m6A-modified *IFNA* and *IFNB* transcripts degrade faster.	Knockout of METTL3 decreases viral protein production. M6A-deficient IAV is less pathogenic in vivo.	[55,65]
SARS-CoV-2 (*Coronaviridae*)	(+)ssRNA	Pos[+]	Low METTL3/14 levels result in weak Type I and III IFN response.	METTL3 KD results in increased RIG-I recognition of viral RNA and up-regulates cytokine/chemokine genes.	[56]
HMPV (*Pneumoviridae*)	(−)ssRNA	Pos[+]	Infection with m6A-deficient virus increases Type I IFN response.	Infection with m6A-deficient virus increase RIG-I expression. RIG-I binds more efficiently to m6A-deficient viral RNA.	[57]
HIV-1 (*Retroviridae*)	(+)ssRNA	Pos[+]	n/a	METTL3/14 KD decreases Rev-RRE interactions and viral RNA export from the nucleus. Host proviral gene transcripts are uniquely methylated upon infection.	[58]
HBV (*Hepadnaviridae*)	dsDNA	Neg[−]	METTL3/14 siRNA increases *IFNB* expression.	M6A negatively affects RIG-I recognition of viral RNA and pathway activation. M6A promotes reverse transcription of viral pgRNA but reduces its half-life and viral protein expression.	[60,63]
KSHV (*Herpesviridae*)	dsDNA	Neg[−]/Pos[+]	n/a	YTHDF2 restricts lytic replication. Host m6A dynamics: during latent infection affect genes with roles in cell transformation; during lytic infection affect genes related to ERK/MAPK, integrin, and hypoxia signaling pathways.	[61,66]
HCMV (*Herpesviridae*)	dsDNA	Pos[+]	METTL14 siRNA increases *IFNB1* expression.	METTL14 siRNA upregulates host genes for innate immunity and inflammation, proliferation, and metabolic control in response to dsDNA.	[62]

**Table 2:** Synthetic and natural compounds targeting RNA-modifying proteins for clinical application in infectious diseases.

Compound	Activity	Model	Target (Function)	Effect on RNA Modification	Ref.
STM2457	IC50: 21 µM	MRC-5 lung fibroblasts infected with HCoV-OC43	METTL3 (inhibition)	Inhibition of m6A formation	[70]
STM2457	IC50: 16.84 µM	A549^+ACE2^ lung carcinoma cells infected with SARS-CoV-2	METTL3 (inhibition)	Inhibition of m6A formation	[70]
Quercetin	IC50: 2.73 µM	In vitro biological activity assay	METTL3–METTL14 (inhibition)	Inhibition of m6A formation	[71]
Luteolin	IC50: 6.23 µM	In vitro biological activity assay	METTL3–METTL14 (inhibition)	Inhibition of m6A formation	[71]
Scutellarin	IC50: 19.93 µM	In vitro biological activity assay	METTL3–METTL14 (inhibition)	Inhibition of m6A formation	[71]
Rhein	200 μg/mL	Vero E6 cell line infected with HCoV-229E, OC43, SARS-CoV-2	m6A demethylase FTO (inhibition)	Increase in RNA m6A level	[72]
3-Deazaadenosine	0.1 mM ED50: (oral) 10 mg/kg/day	Chick embryo cells infected with Rous sarcoma virus; respiratory syncitial (RSV); and parainfluenza type 3 (PI3V) virus in small animal models	S-adenosyl-homocysteine hydrolase (inhibition)	Inhibition of cellular methylation activity	[73,74,75]
Meclofenamic acid	1 μM	BCBL1 cells infected with KSHV	m6A demethylase FTO (inhibition)	Increase in RNA m6A level	[66]
